# *catena*-Poly[[trimethyl­tin(IV)]-μ-1,2,3-benzotriazol-1-ido-κ^2^ *N* ^1^:*N* ^3^]

**DOI:** 10.1107/S1600536811055176

**Published:** 2012-01-18

**Authors:** Yuanyuan Li, Jianmin Liu, Dacheng Li

**Affiliations:** a School of Chemistry and Chemical Engineering, Liaocheng University, Shandong 252059, People’s Republic of China

## Abstract

In the title coordination polymer, [Sn(CH_3_)_3_(C_6_H_4_N_3_)]_*n*_, the Sn^IV^ atom is five-coordinated in a distorted trigonal–bipyramidal geometry with the methyl groups in equatorial positions and two N atoms of two symmetry-related benzotriazolide anions in axial positions. The anion bridges adjacent metal atoms, forming zigzag polymeric chains parallel to [011] and [0
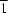
1].

## Related literature

For the biological activity of organotin complexes with nitro­gen donor ligands, see: Pettinari *et al.* (1996 ▶). For related structures, see: Blaschette *et al.* (1992 ▶); Wirth *et al.* (1998 ▶); Berceanc *et al.* (2002 ▶).
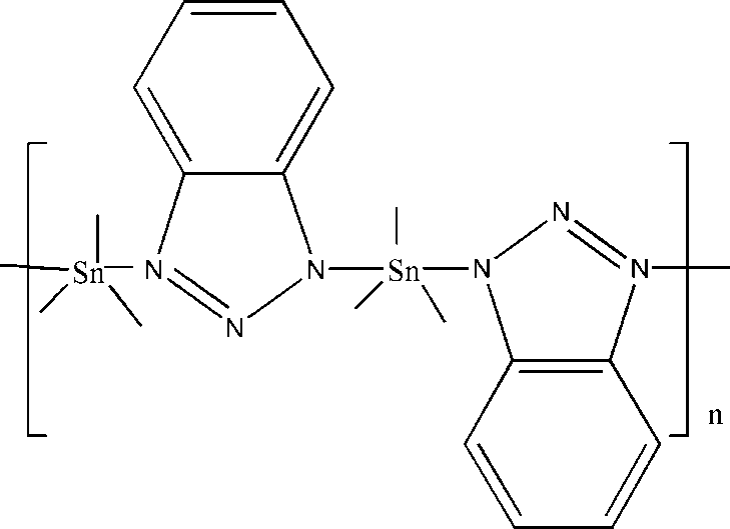


## Experimental

### 

#### Crystal data

[Sn(CH_3_)_3_(C_6_H_4_N_3_)]
*M* *_r_* = 281.93
Orthorhombic, 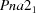

*a* = 14.8168 (14) Å
*b* = 10.6687 (9) Å
*c* = 7.3518 (7) Å
*V* = 1162.14 (18) Å^3^
*Z* = 4
Mo *K*α radiation
μ = 2.16 mm^−1^
*T* = 298 K
0.48 × 0.41 × 0.35 mm

#### Data collection

Bruker SMART 1000 CCD area-detector diffractometer
Absorption correction: multi-scan (*SADABS*; Sheldrick, 2008 ▶) *T*_min_ = 0.424, *T*_max_ = 0.518
5549 measured reflections
1920 independent reflections
1253 reflections with *I* > 2σ(*I*)
*R*_int_ = 0.031

#### Refinement

*R*[*F* ^2^ > 2σ(*F* ^2^)] = 0.030
*wR*(*F* ^2^) = 0.137
*S* = 1.00
1920 reflections
122 parameters
1 restraint
H-atom parameters constrained
Δρ_max_ = 0.49 e Å^−3^
Δρ_min_ = −0.49 e Å^−3^
Absolute structure: Flack (1983 ▶), 801 Fiedel pairs
Flack parameter: −0.11 (13)

### 

Data collection: *SMART* (Siemens, 1996 ▶); cell refinement: *SAINT* (Siemens, 1996 ▶); data reduction: *SAINT*; program(s) used to solve structure: *SHELXS97* (Sheldrick, 2008 ▶); program(s) used to refine structure: *SHELXL97* (Sheldrick, 2008 ▶); molecular graphics: *SHELXTL* (Sheldrick, 2008 ▶); software used to prepare material for publication: *SHELXTL*.

## Supplementary Material

Crystal structure: contains datablock(s) I, global. DOI: 10.1107/S1600536811055176/rz2693sup1.cif

Structure factors: contains datablock(s) I. DOI: 10.1107/S1600536811055176/rz2693Isup2.hkl

Additional supplementary materials:  crystallographic information; 3D view; checkCIF report

## Figures and Tables

**Fig. 1.**
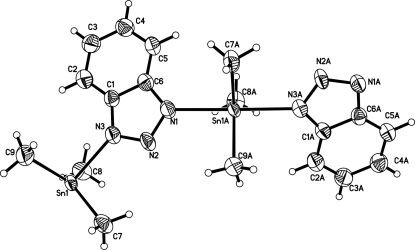
The molecular structure of the title compound with 30% probability displacement ellipsoids. Symmetry code: (A) 1/2-x, 1/2+y, -1/2+z.

## supplementary crystallographic information

### Comment

Several efforts have been made to prepare and study organotin compounds with nitrogen donor ligands not only because of their antitumor activity but also for the important role these compounds play in many biological processes (Pettinari *et al.*, 1996). Since then, only a few such complexes have been reported (Blaschette *et al.*, 1992; Wirth *et al.*, 1998; Berceanc *et al.*, 2002). As a contribution to this field, we report herein the crystal structure of the polymeric title compound.

In the title compound (Fig. 1), the Sn atom displays a distorted trigonal bipyramidal coordination geometry with the equatorial positions occupied by the carbon atoms of the three methyl groups and the apices by the N atoms of two symmetry-related benzotriazolato anion. The Sn—N bond distances are not remarkably different [Sn1—N1^i^ = 2.356 (8); Sn1—N3 = 2.329 (8) Å; symmetry code: (i) 1/2-x, -1/2+y, 1/2-z], with an average value in agreement with those observed in related compounds. In the crystal structure, the benzotriazolato anions link adjacent Sn(Me)_3_ units into zig-zag polymeric chains running parallel to the [011] and [011] direction. Packing is stabilized only by van der Waals forces.

### Experimental

The title compound was prepared by mixing trimethyltin (0.2 g, 1 mmol) and 1,2,3-benzotriazole (0.12 g, 1 mmol) in methanol (10 mL). The mixture was stirred for 7h, then the undissolved substance was filtered off. The title compound crystallized as red crystals after three weeks on slow evaporation of the solvent. Yield: 56%. Anal. Calc (%) for C_18_H_26_N_6_Sn_2_ (563.83): C, 38.31; H, 5.51. Found (%): C, 37.02; H, 5.36.

### Refinement

All H atoms were placed in geometrically idealized positions and treated as riding on their parent atoms, with C—H = 0.93–0.96 Å and with *U*_iso_(H) = 1.2*U*_eq_(C) or 1.5*U*_eq_(C) for methyl H atoms.

### Crystal data

**Table:**

[Sn(CH_3_)_3_(C_6_H_4_N_3_)]	*F*(000) = 552
*M**_r_* = 281.93	*D*_x_ = 1.611 Mg m^−^^3^
Orthorhombic, *P**n**a*2_1_	Mo *K*α radiation, λ = 0.71073 Å
Hall symbol: P 2c -2n	Cell parameters from 2591 reflections
*a* = 14.8168 (14) Å	θ = 2.3–28.1°
*b* = 10.6687 (9) Å	µ = 2.16 mm^−^^1^
*c* = 7.3518 (7) Å	*T* = 298 K
*V* = 1162.14 (18) Å^3^	Block, red
*Z* = 4	0.48 × 0.41 × 0.35 mm

### Data collection

**Table:**

Bruker SMART 1000 CCD area-detector diffractometer	1920 independent reflections
Radiation source: fine-focus sealed tube	1253 reflections with *I* > 2σ(*I*)
graphite	*R*_int_ = 0.031
phi and ω scans	θ_max_ = 25.0°, θ_min_ = 2.4°
Absorption correction: multi-scan (*SADABS*; Sheldrick, 2008)	*h* = −15→17
*T*_min_ = 0.424, *T*_max_ = 0.518	*k* = −12→12
5549 measured reflections	*l* = −7→8

### Refinement

**Table:**

Refinement on *F*^2^	Hydrogen site location: inferred from neighbouring sites
Least-squares matrix: full	H-atom parameters constrained
*R*[*F*^2^ > 2σ(*F*^2^)] = 0.030	*w* = 1/[σ^2^(*F*_o_^2^) + (0.0835*P*)^2^ + 1.6416*P*] where *P* = (*F*_o_^2^ + 2*F*_c_^2^)/3
*wR*(*F*^2^) = 0.137	(Δ/σ)_max_ = 0.002
*S* = 1.00	Δρ_max_ = 0.49 e Å^−^^3^
1920 reflections	Δρ_min_ = −0.49 e Å^−^^3^
122 parameters	Extinction correction: *SHELXL97* (Sheldrick, 2008), Fc^*^=kFc[1+0.001xFc^2^λ^3^/sin(2θ)]^-1/4^
1 restraint	Extinction coefficient: 0.025 (2)
Primary atom site location: structure-invariant direct methods	Absolute structure: Flack (1983), 801 Fiedel pairs
Secondary atom site location: difference Fourier map	Flack parameter: −0.11 (13)

### Fractional atomic coordinates and isotropic or equivalent isotropic displacement parameters (Å^2^)

**Table:**

	*x*	*y*	*z*	*U*_iso_*/*U*_eq_	
N1	0.1848 (6)	0.4850 (7)	0.0322 (11)	0.065 (2)	
Sn1	0.25035 (3)	0.15544 (4)	0.3757 (6)	0.0479 (3)	
C4	−0.0362 (7)	0.3651 (9)	−0.123 (2)	0.081 (3)	
H4	−0.0862	0.3824	−0.1949	0.097*	
C5	0.0335 (6)	0.4485 (8)	−0.120 (2)	0.075 (3)	
H5	0.0331	0.5201	−0.1917	0.090*	
N3	0.1867 (5)	0.3116 (7)	0.1954 (13)	0.067 (2)	
C6	0.1055 (7)	0.4217 (8)	−0.0033 (13)	0.061 (2)	
C7	0.3625 (7)	0.2679 (9)	0.4423 (16)	0.079 (3)	
H7A	0.4164	0.2180	0.4382	0.118*	
H7B	0.3549	0.3014	0.5625	0.118*	
H7C	0.3673	0.3354	0.3565	0.118*	
C1	0.1073 (7)	0.3123 (8)	0.0980 (14)	0.060 (2)	
C9	0.1374 (7)	0.1572 (11)	0.555 (2)	0.102 (5)	
H9A	0.1208	0.2423	0.5812	0.152*	
H9B	0.1530	0.1152	0.6662	0.152*	
H9C	0.0875	0.1149	0.4987	0.152*	
N2	0.2332 (6)	0.4168 (8)	0.1502 (15)	0.067 (2)	
C8	0.2505 (6)	0.0421 (12)	0.139 (2)	0.076 (4)	
H8A	0.3055	0.0554	0.0726	0.115*	
H8B	0.2000	0.0640	0.0637	0.115*	
H8C	0.2462	−0.0445	0.1732	0.115*	
C2	0.0372 (9)	0.2279 (11)	0.0931 (19)	0.085 (4)	
H2	0.0384	0.1557	0.1639	0.102*	
C3	−0.0351 (10)	0.2533 (11)	−0.020 (2)	0.088 (4)	
H3	−0.0828	0.1969	−0.0286	0.106*	

### Atomic displacement parameters (Å^2^)

**Table:**

	*U*^11^	*U*^22^	*U*^33^	*U*^12^	*U*^13^	*U*^23^
N1	0.074 (5)	0.059 (5)	0.062 (6)	0.000 (4)	0.001 (4)	0.014 (4)
Sn1	0.0570 (4)	0.0380 (4)	0.0489 (4)	0.0003 (2)	0.0018 (3)	0.0109 (3)
C4	0.083 (7)	0.084 (7)	0.075 (7)	0.001 (6)	−0.009 (9)	0.004 (8)
C5	0.082 (6)	0.068 (6)	0.075 (7)	0.001 (5)	0.004 (9)	0.020 (7)
N3	0.073 (5)	0.058 (5)	0.069 (6)	0.001 (4)	0.001 (5)	0.013 (4)
C6	0.073 (6)	0.057 (5)	0.054 (6)	0.005 (5)	0.003 (5)	0.001 (5)
C7	0.097 (7)	0.054 (6)	0.086 (8)	0.002 (5)	−0.018 (6)	0.012 (5)
C1	0.067 (6)	0.053 (5)	0.060 (7)	−0.005 (5)	−0.002 (5)	0.004 (5)
C9	0.083 (8)	0.103 (10)	0.119 (12)	0.018 (6)	0.022 (9)	0.042 (8)
N2	0.087 (6)	0.040 (4)	0.073 (7)	−0.001 (4)	−0.009 (5)	0.015 (4)
C8	0.096 (9)	0.053 (6)	0.080 (9)	−0.003 (4)	−0.020 (6)	−0.018 (5)
C2	0.087 (7)	0.073 (7)	0.094 (10)	−0.005 (6)	−0.002 (8)	0.018 (7)
C3	0.078 (7)	0.075 (8)	0.112 (11)	−0.010 (6)	−0.009 (8)	0.008 (7)

### Geometric parameters (Å, °)

**Table:**

N1—N2	1.340 (11)	C6—C1	1.385 (12)
N1—C6	1.380 (12)	C7—H7A	0.9600
N1—Sn1^i^	2.356 (8)	C7—H7B	0.9600
Sn1—C7	2.107 (10)	C7—H7C	0.9600
Sn1—C8	2.120 (13)	C1—C2	1.375 (13)
Sn1—C9	2.131 (13)	C9—H9A	0.9600
Sn1—N3	2.329 (8)	C9—H9B	0.9600
Sn1—N1^ii^	2.356 (8)	C9—H9C	0.9600
C4—C5	1.364 (12)	C8—H8A	0.9600
C4—C3	1.411 (16)	C8—H8B	0.9600
C4—H4	0.9300	C8—H8C	0.9600
C5—C6	1.397 (15)	C2—C3	1.384 (19)
C5—H5	0.9300	C2—H2	0.9300
N3—N2	1.359 (11)	C3—H3	0.9300
N3—C1	1.376 (12)		
			
N2—N1—C6	108.2 (7)	H7A—C7—H7B	109.5
N2—N1—Sn1^i^	121.1 (6)	Sn1—C7—H7C	109.5
C6—N1—Sn1^i^	129.2 (6)	H7A—C7—H7C	109.5
C7—Sn1—C8	121.0 (5)	H7B—C7—H7C	109.5
C7—Sn1—C9	118.1 (6)	N3—C1—C2	131.0 (9)
C8—Sn1—C9	120.9 (5)	N3—C1—C6	107.5 (8)
C7—Sn1—N3	92.5 (3)	C2—C1—C6	121.5 (10)
C8—Sn1—N3	86.6 (5)	Sn1—C9—H9A	109.5
C9—Sn1—N3	91.6 (4)	Sn1—C9—H9B	109.5
C7—Sn1—N1^ii^	90.3 (4)	H9A—C9—H9B	109.5
C8—Sn1—N1^ii^	87.7 (5)	Sn1—C9—H9C	109.5
C9—Sn1—N1^ii^	91.4 (4)	H9A—C9—H9C	109.5
N3—Sn1—N1^ii^	174.3 (4)	H9B—C9—H9C	109.5
C5—C4—C3	122.3 (11)	N1—N2—N3	109.6 (8)
C5—C4—H4	118.9	Sn1—C8—H8A	109.5
C3—C4—H4	118.9	Sn1—C8—H8B	109.5
C4—C5—C6	117.1 (10)	H8A—C8—H8B	109.5
C4—C5—H5	121.5	Sn1—C8—H8C	109.5
C6—C5—H5	121.5	H8A—C8—H8C	109.5
N2—N3—C1	107.6 (8)	H8B—C8—H8C	109.5
N2—N3—Sn1	121.6 (6)	C3—C2—C1	118.2 (11)
C1—N3—Sn1	130.2 (6)	C3—C2—H2	120.9
N1—C6—C1	107.1 (9)	C1—C2—H2	120.9
N1—C6—C5	131.8 (9)	C2—C3—C4	119.7 (12)
C1—C6—C5	121.1 (9)	C2—C3—H3	120.1
Sn1—C7—H7A	109.5	C4—C3—H3	120.1
Sn1—C7—H7B	109.5		
			
C3—C4—C5—C6	−2.8 (17)	N2—N3—C1—C6	−1.6 (12)
C7—Sn1—N3—N2	10.1 (9)	Sn1—N3—C1—C6	−173.1 (7)
C8—Sn1—N3—N2	−110.8 (9)	N1—C6—C1—N3	0.9 (11)
C9—Sn1—N3—N2	128.3 (9)	C5—C6—C1—N3	178.9 (10)
C7—Sn1—N3—C1	−179.4 (10)	N1—C6—C1—C2	179.8 (11)
C8—Sn1—N3—C1	59.7 (9)	C5—C6—C1—C2	−2.2 (16)
C9—Sn1—N3—C1	−61.2 (10)	C6—N1—N2—N3	−1.2 (12)
N2—N1—C6—C1	0.1 (11)	Sn1^i^—N1—N2—N3	−168.6 (7)
Sn1^i^—N1—C6—C1	166.2 (7)	C1—N3—N2—N1	1.7 (12)
N2—N1—C6—C5	−177.5 (11)	Sn1—N3—N2—N1	174.1 (6)
Sn1^i^—N1—C6—C5	−11.5 (16)	N3—C1—C2—C3	−179.8 (13)
C4—C5—C6—N1	−179.9 (10)	C6—C1—C2—C3	1.6 (19)
C4—C5—C6—C1	2.7 (15)	C1—C2—C3—C4	−2(2)
N2—N3—C1—C2	179.6 (12)	C5—C4—C3—C2	2(2)
Sn1—N3—C1—C2	8.2 (19)		

Symmetry codes: (i) −*x*+1/2, *y*+1/2, *z*−1/2; (ii) −*x*+1/2, *y*−1/2, *z*+1/2.

## References

[bb1] Berceanc, V., Crainic, C., Haiduc, I., Mahon, M. F., Molloy, K. C., Venter, M. M. & Wilson, P. J. (2002). *J. Chem. Soc. Dalton Trans.* pp. 1036–1045.

[bb2] Blaschette, A., Hippel, I., Krahl, J., Wieland, E., Jones, P. G. & Sebald, A. (1992). *J. Organomet. Chem.* **437**, 279–297.

[bb3] Flack, H. D. (1983). *Acta Cryst.* A**39**, 876–881.

[bb4] Pettinari, C., Marchetti, F., Pellei, M., Cingolani, A., Barba, L. & Cassetta, A. (1996). *J. Organomet. Chem.* **515**, 119–130.

[bb5] Sheldrick, G. M. (2008). *Acta Cryst.* A**64**, 112–122. 10.1107/S0108767307043930 18156677

[bb6] Siemens (1996). *SMART* and *SAINT* Siemens Analytical X-ray Instruments Inc., Madison, Wisconsin, USA.

[bb7] Wirth, A., Lange, I., Henschel, D., Moers, O., Blaschette, A. & Jones, P. G. (1998). *Z. Anorg. Allg. Chem.* **624**, 1308–1318.

